# Stone or Gravel

**Published:** 1875-01

**Authors:** 


Stone or Gravel.—Dwellers in localities where the well-water is hard, that is, where it contains considerable carbonate of lime, are aware that the tea-kettle, or other vessel in which water is boiled, speedily becomes coated with a thick limy deposit, which is precipitated from the water at the boiling point. All good housewives know that to boil a mess of potatoes in a vessel thus coated, will remove the incrustation. In like manner the furring or coating deposited on the inside of steam boilers may be easily removed, making the surface appear like new iron, by placing a quantity of raw potatoes in the boiler and letting them boil to pieces. After two or three days, open the manholes, and a sandy deposit will be found ; brush it out and the boilers will be good as new. Acting on this hint, it has been ascertained, as a trustworthy correspondent assures us, that the worst case of "gravel," or stone in the bladder may be cured, the calcareous deposit dissolved and passed away, by using the water in which potatoes have boiled to pieces. Strain the water, sweeten to taste, and drink copiously for two or three weeks, using no other beverage, meantime, but new milk. Its action is purely chemical and can do no harm.

